# Etymologia: Varicella Zoster Virus

**DOI:** 10.3201/eid2104.ET2104

**Published:** 2015-04

**Keywords:** etymologia, varicella zoster virus, viruses, variola, chickenpox, herpes zoster, shingles

## Varicella Zoster Virus [var″i-sel′ə zos′tər vi′rəs]

A member of the family *Herpesviridae*, varicella zoster virus (VZV) is named for the 2 main diseases (chickenpox and herpes zoster [shingles]) it causes. Varicella (Figure) may be a diminutive of “variola” because it was considered a mild form of smallpox. “Variola” was coined by Rudolph Augustin Vogel in 1764 and is possibly derived from the Latin *varus* (“pimple”) or *varius *(“speckled”). Herpes zoster derives from the Greek terms *herpein* (“to creep”) and *zoster* (“belt”). Not until the twentieth century was VZV recognized as the cause of both these diseases.

**Figure:**
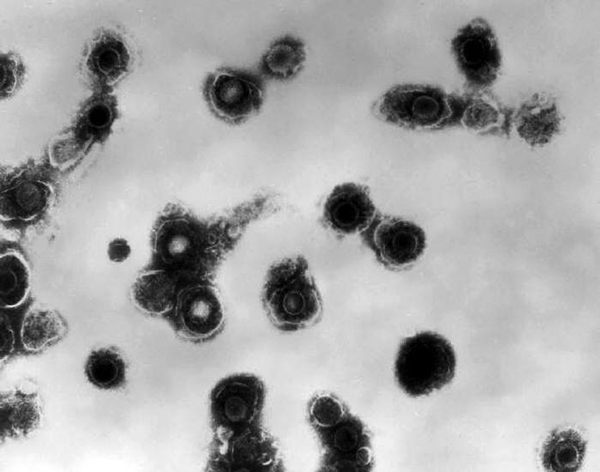
Legend: Electron micrograph showing varicella zoster virus.

## References

[1] Lustig R. Dictionary of medical terminology [in Italian]. Milan: Società Editrice Libraria; 1927. p. 495.

[2] Weller TH. Historical perspective. In: Arvin AM, Gershon AA, editors. Varicella-zoster virus: virology and clinical management. Cambridge (UK): Cambridge University Press; 2000. p. 9–24.

[3] Weller TH. Varicella: historical perspective and clinical overview. J Infect Dis. 1996;174(Suppl 3):S306–9 . 10.1093/infdis/174.Supplement_3.S306 8896536

